# Impact of pre-treatment prognostic nutritional index and the haemoglobin, albumin, lymphocyte and platelet (HALP) score on endometrial cancer survival: A prospective database analysis

**DOI:** 10.1371/journal.pone.0272232

**Published:** 2022-08-04

**Authors:** Kelechi Njoku, Chloe E. Barr, Neal C. Ramchander, Emma J. Crosbie

**Affiliations:** 1 Division of Cancer Sciences, Faculty of Biology, Medicine and Health, School of Medical Sciences, St Mary’s Hospital, University of Manchester, Manchester, United Kingdom; 2 Stoller Biomarker Discovery Centre, Institute of Cancer Sciences, Faculty of Biology, Medicine and Health, University of Manchester, Manchester, United Kingdom; 3 Department of Obstetrics and Gynaecology, Manchester University NHS Foundation Trust, Manchester Academic Health Science Centre, Manchester, United Kingdom; University of Campinas, BRAZIL

## Abstract

**Purpose:**

The Onodera’s prognostic nutritional index (PNI) and the haemoglobin, albumin, lymphocyte and platelet (HALP) score are immune-nutritional indices that correlate with survival outcomes in several adult solid malignancies. The aim of this study was to investigate whether PNI and HALP are associated with survival outcomes in endometrial cancer.

**Patients and methods:**

Women undergoing management for endometrial cancer were recruited to a single centre prospective cohort study. Pre-treatment PNI and HALP scores were computed for study participants and analysed as continuous variables and by selecting cut-off values based on previous publications. Both parameters were analysed in relation to overall, endometrial cancer-specific and recurrence-free survival using Kaplan-Meier estimation and multivariable Cox proportional regression.

**Results:**

A total of 439 women, with a median age of 67 years (interquartile range (IQR), 58, 74) and BMI of 31kg/m^2^ (IQR 26, 37) were included in the analysis. Most had low-grade (63.3%), early-stage (84.4% stage I/II) endometrial cancer of endometrioid histological subtype (72.7%). Primary treatment was surgery in 98.2% of cases. Adjusted overall mortality hazard ratios for PNI and HALP as continuous variables were 0.97(95%CI 0.94–1.00, p = 0.136) and 0.99(95%CI 0.98–1.01, p = 0.368), respectively. Women with pre-treatment PNI ≥45 had a 45% decrease in both overall (adjusted HR = 0.55, 95% CI 0.33–0.92, p = 0.022) and cancer-specific mortality risk (adjusted HR = 0.55, 95%CI 0.30–0.99, p = 0.048) compared to those with PNI <45. There was no evidence for an effect of PNI on recurrence free survival. HALP scores were associated with adverse clinico-pathologic factors, but not overall, cancer-specific or recurrence-free survival in the multivariable analysis.

**Conclusion:**

PNI is an independent prognostic factor in endometrial cancer and has the potential to refine pre-operative risk assessment.

## Introduction

Endometrial cancer, the leading gynaecological malignancy in high income countries, affects over 400,000 women every year globally and its incidence is rising alongside growing rates of obesity [1, 2]. In the United Kingdom (UK), it is the fourth most common cancer affecting women with approximately 9400 new diagnoses and 2500 deaths from the disease in 2018 [3]. Although most women with endometrial cancer are diagnosed with disease that is amenable to curative surgical resection, a significant minority present with advanced or metastatic disease and face a dismal prognosis [2, 4]. Identifying factors that influence survival is crucial to mitigating the impact of the rising disease burden [5, 6].

Nutritional status and host immunity correlate with survival outcomes in several adult solid malignancies [7–12]. Cancer-associated malnutrition is commonly associated with a deceased immune function and predisposes to increased post-operative morbidity and mortality [8]. Impaired immune-nutritional status in patients with cancer can result in delayed wound healing, poor tolerance and response to chemotherapy, prolonged hospital stay and poor outcomes [7, 13]. The impact of pre-operative immuno-nutritional status on treatment outcomes has been explored in several malignancies including ovarian [9] and cervical cancer [14, 15], although endometrial cancer remains relatively understudied [6].

The prognostic nutritional index (PNI) is a composite measure of immune-nutritional status and is based on a linear predictive model that incorporates pre-operative serum albumin and total lymphocyte counts [16]. A low PNI has been shown to be a negative prognostic factor in cancers of the ovary [17, 18], cervix [14, 15], lung [10], colon [19] and pancreas [20]. A meta-analysis of 2373 patients showed that PNI was significantly associated with overall and progression-free survival in patients with gynaecological malignancies, but no endometrial cancer studies were included in this review [21]. The haemoglobin, albumin, lymphocyte and platelet (HALP) score, a comprehensive index of nutrition and systemic inflammation is a promising cancer prognostic biomarker [22]. HALP may also reflect cancer-related anaemia, a likely consequence of advanced malignancy, nutritional deficiency and chronic blood loss [23]. To our knowledge, this is the first study exploring the potential prognostic utility of the HALP score in endometrial cancer.

The aim of this study was to investigate whether pre-treatment PNI and HALP scores are associated with survival outcomes using a large prospective database of endometrial cancer patients.

## Methods

### Ethics and approvals

This study uses data from a prospectively maintained database of women with endometrial cancer, participating in clinical research at St Mary’s Hospital, Manchester. All women gave written, informed consent for their pseudo-anonymised clinical data to be used in future research. The primary research studies were: Metformin (North West Research Ethics Committee, NW REC, 11/NW/0442, approved 19 August 2011), Weight loss (NW REC, 12/NW/0050, approved 23 January 2012), PREMIUM (NW REC, 14/NW/1236, approved 23 September 2014), PETALS (NRES Committee North West, Lancaster, 15/NW/0733, approved 18 September 2015) and DETECT (NW REC, Greater Manchester, 16/NW/0660, approved 16 September 2016).

### Study population

We recruited women diagnosed with endometrial cancer between 2010 and 2016 at St Mary’s Hospital, Manchester, a regional specialist centre for the management of gynaecological malignancies, to a prospective cohort study. We collected relevant sociodemographic and clinico-pathological data at baseline, including age, body mass index (BMI), comorbidities, socioeconomic quintile, histological subtype, tumour grade and stage, percentage depth of myometrial invasion and the presence of lymphovascular space invasion (LVSI). We classified women as underweight (BMI<18.5 kg/m^2^), normal weight (BMI 18.5–24.9kg/m^2^), overweight (BMI 25–29.9kg/m^2^) or obese (BMI≥30kg/m^2^) based on the World Health Organization (WHO) BMI categories. We categorised participants into two age groups (<65 and ≥65 years), in line with age groupings used in many studies. Endometrial cancers were classified according to histological subtype (endometrioid, serous, clear cell, carcinosarcoma) based on review by two specialist gynaecological pathologists, reporting to the Royal College of Pathology Standards and in line with the revised FIGO (International Federation of Gynecology and Obstetrics) 2009 surgical staging criteria [24]. The primary treatment for most women was total hysterectomy and bilateral salpingo‐oophorectomy. Adjuvant therapy was offered to those with intermediate and high risk disease in accordance with international guidelines [25, 26]. A few women with grade 1 stage 1a endometrial cancer who wished to preserve their fertility, or who were medically unfit for surgery, received primary hormone therapy (+/-delayed hysterectomy).

All patients were reviewed in routine follow-up clinics at 3‐month (for 3 years), 6‐month (for 1 year) and 12‐month intervals for a total period of 5 years, or until recurrence or death, whichever was sooner. General practitioners were contacted at regular intervals in order to ascertain current status in cases of women who had completed routine hospital-based follow up or moved away from the study area. Women with disease recurrence were managed according to international recommendations [26]. Those with pelvic recurrence were managed by surgery or radiotherapy as appropriate, whereas those with metastatic, inoperable pelvic or distant recurrence were managed with palliative hormone therapy, chemotherapy or radiotherapy [27]. We obtained cause of death information from death certificates.

### Immuno-nutritional indices

We measured pre-treatment complete blood count (CBC) and albumin levels for the study participants. PNI was calculated as 10 × serum albumin (g/dl) + 0.005 × total lymphocyte count (per mm^3^). HALP was calculated as haemoglobin (g/L) × albumin (g/L) levels × lymphocyte count (/L)/platelet count (/L). The main study endpoints were overall, cancer-specific and recurrence-free survival.

### Statistical analysis

Overall survival was defined as the duration from the date of primary treatment to death from any cause or the last day of follow-up. Cancer‐specific survival was calculated from date of primary treatment to death from endometrial cancer or the date of last follow-up and censored at the date of death from other causes. Recurrence‐free survival was calculated from the date of primary treatment to disease recurrence, death or date of last follow-up, whichever was sooner. PNI and HALP were analysed as continuous variables and by selecting cut-off values based on previous publications [9, 16, 28–30]. For PNI, a cut-off of 45 was identified as the most commonly reported prognostic threshold in gynaecological malignancies [21], and a cut-off of 24 for HALP [31]. The associations between PNI, HALP and clinico-pathological characteristics were tested using Chi-square (X^2^) and Fisher’s exact tests, as appropriate. The Kaplan–Meier method and log-rank test were used to compare survival between prognostic categories. Cox regression multivariable analysis was used to model the association between the nutritional parameters and survival while adjusting for confounding and effect modifications. Hazard ratios (HRs) with 95% confidence intervals (95% CIs) were presented for both univariable and multivariable analyses. The confounding factors included in the models were age at diagnosis, BMI, socioeconomic quintile, type 2 diabetes mellitus (T2DM) status, treatment modality, FIGO stage, histological subtype, grade, lymphovascular space invasion (LVSI), and depth of myometrial invasion, as well-established prognostic indicators. Confounding was assessed by the changes in the hazard coefficients following the introduction of these variables to the regression models. Two-tailed *P*-values <0.05 were considered statistically significant. All analyses were conducted using the statistical package Stata 16.0 (**https://www.stata.com**).

## Results

### Descriptive characteristics of the study population

In total, 439 histologically confirmed endometrial cancer patients with a median age of 67 years (interquartile range (IQR), 58, 74) and BMI of 31kg/m^2^ (IQR 26, 37) were included in the analysis. The socio-demographic and clinico-pathological characteristics of the study participants are summarized in Table 1. Most of the included patients were ≥65 years of age (58.9%), overweight or obese (81.8%) and had low-grade (63.3%), early-stage (84.4% stage I/II) endometrial cancer of endometrioid histology (72.7%). The modal social deprivation quintile was quintile 1(most deprived), accounting for 35.8% of the study population, and 18.8% had type 2 diabetes mellitus. Surgery was the primary treatment in 98.2% of patients and 49.1% received adjuvant therapy. Over the study period, 68 women (15.5%) relapsed, 93 (21.2%) died, and the remainder were alive as of 31 March 2021.

**Table 1:** Socio-demographic characteristics of the study population.

Variable	n (% total)
**Age at diagnosis**	Median age 67 years (IQR 58, 74)
<65 years	181 (41.2%)
≥65 years	258 (58.9%)
**Body Mass Index (kg/m** ^**2**^ **)**	Median BMI 31kg/m^2^ (IQR 26, 37)
Underweight	5 (1.1%)
Normal weight	75 (17.1%)
Overweight	117 (26.7%)
Obese	242 (55.1%)
**Tumour grade**	
1	178 (40.1%)
2	102 (23.2%)
3	159 (36.2%)
**FIGO stage**	
I	323 (73.7%)
II	47 (10.7%)
III	61 (13.9%)
IV	7 (1.6%)
**Histology**	
Endometrioid	319 (72.7%)
Non-endometrioid	120 (27.3%)
**Lymphovascular space invasion**	
No	297 (68.0%)
Yes	140 (32.0%)
**Depth of myometrial invasion**	
<50%	273 (62.2%)
≥50%	166 (37.8%)
**Social deprivation quintile**	
Quintile I (Most deprived)	157 (35.8%)
Quintile II	106 (24.2%)
Quintile III	50 (11.4%)
Quintile IV	76 (17.3%)
Quintile V (Least deprived)	50 (11.4%)
**History of type 2 diabetes mellitus (n = 535)**	
Yes	82 (18.8%)
No	354 (81.2%)
**Primary treatment**	
Surgery	431 (98.2%)
Hormonal (Fertility sparing reasons)	2 (0.5%)
Hormonal (Not fit for surgery)	5 (1.1%)
Radiotherapy	1 (0.2%)
**Adjuvant treatment**	
Yes	215 (49.1%)
No	223 (50.9%)
**Recurrence**	
Yes	68 (15.5%)
No	371 (84.5%)
**Survival status at end of follow up**	
Alive	346 (78.8%)
Cancer-specific mortality	67 (15.3%)
Non-cancer related mortality	26 (5.9%)
**Total**	**439 (100%)**

### Kaplan-Meier survival estimation and Cox regression analysis

Women were followed up for a median duration of 42 months (IQR 27–59 months). The overall survival rates for the study cohort were 95% (93–97%) at 12 months, 85% (81–88%) at 36 months and 76% (71–81%) at 60 months. Univariate Cox regression analysis confirmed age, T2DM, FIGO stage, disease grade, histology, presence of (LVSI) and depth of myometrial invasion as important predictors of overall survival. For every unit increase in age, there was a 6% increase in overall mortality risk (HR 1.06, 95%CI 1.04–1.09, p<0.001). Women with advanced disease (FIGO stages III/IV) had a near three-fold higher mortality risk (HR 2.74, 95%CI 1.75–4.30, p<0.001) than those with early stage disease (FIGO stages I/II), whereas those with comorbid diabetes mellitus had a 76% increased risk of death (HR 1.76, 95% CI 1.12–2.76, p = 0.014) compared to those without. There was no evidence of an effect of BMI on overall mortality (HR per unit increase in BMI = 0.99, 95%CI 0.97–1.02, p = 0.636). Compared with women with low-grade disease (grades I/II), those with high-grade disease (grade III) had a three-fold higher risk of death (HR 3.16, 95% CI 2.07–4.81, p<0.001), while those with endometrioid cancer were also at a three-fold increased risk of death (HR 3.27, 95% 2.16–4.93, p<0.001) compared to non-endometrioid tumours. The presence of LVSI and deep myometrial invasion also correlated with higher overall mortality risks (HR = 2.29, 95% CI 1.52–3.44, p<0.001 and HR = 1.84, 95% CI 1.22–2.77, p = 0.003, respectively).

Over the study period, there were 93 deaths, of which 67 (72.0%) were due to endometrial cancer. Cancer-specific survival was 96% (94–98%) at 12 months, 89% (85–91%) at 36 months and 81% (76–86%) at 60 months. Univariate Cox regression revealed that age at diagnosis (HR = 1.05, 95%CI 1.03–1.08, p<0.001), FIGO stage (HR = 4.31, 95%CI 2.63–7.07, p<0.001), grade (HR = 5.27, 95%CI 3.06–9.07, p<0.001), histology (HR = 4.82, 95%CI 2.93–7.94, p<0.001), LVSI (HR = 3.12 95%CI 1.92–5.07, p<0.001) and deep myometrial invasion (HR = 2.08, 95%CI 1.29–3.37, p = 0.003) all had significant associations with cancer-specific survival. Overall, 68 women relapsed during the study period, with a median time to recurrence of 14 months (range 1–54 months). The recurrence-free survival for the study cohort was 93% (90–95%) at 12 months, 82% (78–86%) at 36 months and 79% (74–83%) at 60 months. The univariable Cox regression analyses confirmed age at diagnosis (HR = 1.04, 95%CI 1.02–1.07, p = 0.001), FIGO stage (HR = 4.31, 95%CI 2.64–7.05, p<0.001), grade (HR = 4.32, 95%CI 2.60–7.19, p<0.001), histology (HR = 3.52, 95%CI 2.19–5.68, p<0.001), LVSI (HR = 3.37, 95%CI 2.08–5.46, p<0.001), deep myometrial invasion (HR = 2.07, 95%CI 1.29–3.34, p = 0.003) and T2DM status (HR = 1.78, 95%CI 1.05–3.03, p = 0.032) as important predictors of recurrence-free survival.

### Pre-treatment PNI and endometrial cancer overall, cancer-specific and recurrence-free survival

PNI values ranged from 32.1 to 66.4 with a median PNI value of 50.1 and IQR of 46.3–53.9. There was no evidence of a correlation between PNI and age (Spearman correlation coefficient -0.07, p = 0.1510), or BMI (Spearman correlation coefficient -0.01, p = 0.862). A PNI cut-off value of 45 was identified as the most commonly reported threshold in gynaecological malignancies based on previous publications [21]. A total of 83 women (18.9%) with PNI values <45 were classed as having ‘low’ PNI whilst the remaining 356 with PNI ≥45 were classed as having ‘high’ PNI. There was an association between PNI and FIGO stage (p = 0.035), myometrial invasion (p = 0.030), primary treatment (p = 0.045) and alive status (p = 0.027) (Table 2). There was no evidence for an association with age, BMI, socioeconomic status, histology, grade or LVSI at the prognostic cut-off of 45 (Table 2).

**Table 2:** Socio-demographic characteristics stratified by prognostic nutritional index (PNI) and haemoglobin, albumin, lymphocyte and platelet (HALP) score categories.

		Frequency	PNI < 45	PNI ≥ 45	p value	HALP<24	HALP≥24	p value
Age (years)	<65	181	33(18.2%)	148(81.8%)	0.762	36(19.9%)	145(80.1%)	0.456
	≥65	258	50(19.4%)	208(80.6%)		59(22.9%)	199(77.1%)	
BMI (kg/m^2^)	Underweight	5	1(20.0%)	4(80.0%)	0.866	2(40.0%)	3(60.0%)	0.141
	Normal	75	16(21.3%)	59(78.7%)		22(29.3%)	53(70.7%)	
	Overweight	117	20(17.1%)	97(82.9%)		27(23.1%)	90(76.9%)	
	Obese	242	46(19.0%)	196(81.0%)		44(18.2%)	198(81.8%)	
FIGO stage	I	323	57(17.6%)	266(82.4%)	**0.035**	58(18.0%)	265(82.0%)	**0.005**
	II	47	12(25.5%)	35(74.5%)		14(29.8%)	33(70.2%)	
	III	61	10(16.4%)	51(83.6%)		19(31.2%)	42(68.8%)	
	IV	7	4(57.1%)	3(42.9%)		4(57.1%)	3(42.9%)	
Histology	Endometrioid	319	55(17.2%)	264(82.8%)	0.146	61(19.1%)	258(80.9%)	**0.037**
	Others	120	28(23.3%)	92(76.7%)		34(28.3%)	86(71.7%)	
Grade	I	178	30(16.8%)	148(83.2%)	0.207	31(17.4%)	147(82.6%)	**0.020**
	II	102	16(15.7%)	86(84.3%)		18(17.7%)	84(82.3%)	
	III	159	37(23.3%)	122(76.7%)		46(28.9%)	113(71.1%)	
LVSI	No	297	51(17.2%)	246(82.8%)	0.214	55(18.5%)	242(81.5%)	0.027
	Yes	140	31(22.1%)	109(77.9%)		39(27.9%)	101(72.1%)	
Myometrial invasion	<50%	273	43(15.8%)	230 (84.2%)	**0.030**	45(16.5%)	228(83.5.5%)	**0.001**
	≥50%	166	40(24.1%)	126(75.9%)		50(30.1%)	116(69.9%)	
Type 2 diabetes mellitus	No	354	68(19.2%)	286(80.8%)	0.656	78(22.0%)	276(78.0%)	0.617
	Yes	82	14(17.1%)	68(82.9%)		16(19.5%)	66(80.5%)	
Social quintile	I	157	34(21.7%)	123(78.3%)	0.185	36(22.9%)	121(77.1%)	0.504
	II	106	18(17.0%)	88(83.0%)		25(23.6%)	81(76.4%)	
	III	50	10(20.0%)	40(80.0%)		10(20.0%)	40(80.0%)	
	IV	76	8(10.5%)	68(89.5%)		11(14.5%)	65(85.5%)	
	V	50	13(26.0%)	37(74.0%)		13(26.0%)	37(74.0%)	
Primary Treatment	Surgery	431	79(18.3%)	352(81.7%)	**0.045**	94(21.8%)	337(78.2%)	**0.776**
	Hormonal	7	4(57.1%)	3(42.9%)		1(14.3%)	6(85.7%)	
	Radiotherapy	1	0(0.0%)	1(100.0%)		0(0.0%)	1(100.0%)	
Adjuvant therapy	No	223	42(18.8%)	181(81.2%)	0.950	44(19.7%)	179(80.3%)	0.311
	Yes	215	41(19.1%)	174(80.9%)		51 (23.7%)	164(76.3%)	
Recurrence	No	371	66(17.8%)	305(82.2%)	0.163	74(20.0%)	297(80.0%)	**0.044**
	Yes	68	17(25.0%)	51(75.0%)		21 (30.9%)	47 (69.1%)	
Alive status	No	93	25(26.9%)	68(73.1%)	**0.027**	28(30.1%)	65(69.9%)	**0.026**
	Yes	346	58(16.8%)	288(83.2%)		67(19.4%)	279 (80.6%)	

Next, we evaluated the performance of PNI as a continuous variable in predicting overall, cancer-specific and recurrence-free survival. There was a 5% reduction in overall mortality per unit increase in PNI (unadjusted HR 0.95, 95% CI 0.91–0.98, p = 0.004). Following adjustment for clinico-pathological parameters, there was 3% reduction in overall mortality per unit increase in PNI, although this was not statistically significant (adjusted HR 0.97, 95% CI 0.94–1.00, p = 0.136). For cancer-specific mortality, the unadjusted and adjusted hazard ratios were 0.95(95% CI 0.91–0.99, p = 0.016) and 0.98(0.94–1.02, p = 0.347), respectively while for disease recurrence, the hazard ratios were 0.98(95%CI 0.94–1.02, p = 0.364) and 1.00(95%CI 0.97–1.05, p = 0.702), respectively.

**Fig 1:**
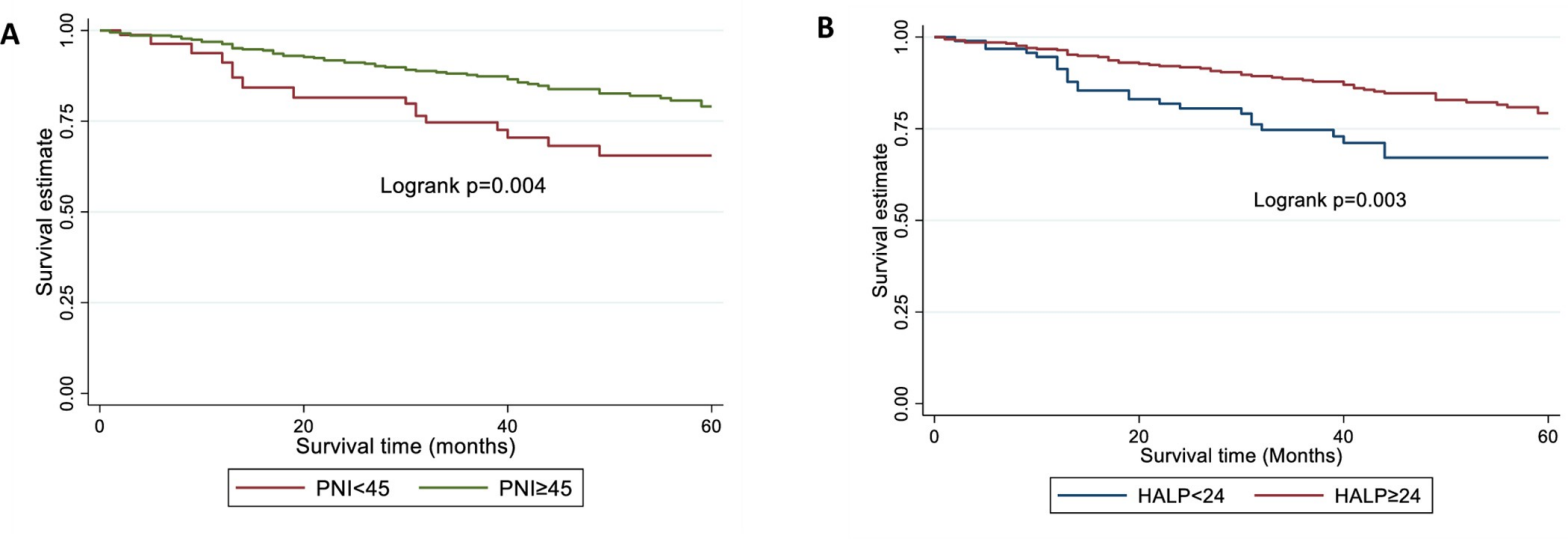
Kaplan-Meier survival curves showing overall survival estimates by prognostic categories in women with endometrial cancer. (A) PNI (B) HALP. There was no evidence for an effect of pre-treatment PNI on recurrence free survival.

Next, we assessed the performance of PNI prognostic categories based on the cut-off of 45. The 5-year survival rates were 66% (52–76%) and 78% (72–83%) for women with low and high PNI values respectively. Women with high PNI had a 45% decrease in overall (adjusted HR 0.55, 95% CI 0.33–0.92, p = 0.022) and cancer-specific (adjusted HR 0.55, 95% CI 0.30–0.99, p = 0.048) mortality risk compared to those with low PNI in multivariable analyses (Fig 1A, Table 3).

**Table 3:** Cox regression analyses of pre-treatment prognostic nutritional index (PNI) and endometrial cancer survival outcomes with crude and adjusted hazard ratios and 95% confidence intervals.

PNI Categories	Unadjusted HR (95%CI)	p-value	Adjusted HR (95%CI)	p-value
**Overall Mortality**				
PNI <45	1.00		1.00	
PNI ≥45	0.51 (0.32–0.81)	**0.004**	0.55 (0.33–0.92)	**0.022**
**Cancer-Specific Mortality**				
PNI <45	1.00		1.00	
PNI ≥45	0.50 (0.29–0.86)	**0.012**	0.55 (0.30–0.99)	**0.048**
**Disease Recurrence**				
PNI <45	1.00		1.00	
PNI ≥45	0.61(0.35–1.06)	0.080	0.61 (0.34–1.12)	0.111

Adjusted model includes age, BMI, socioeconomic quintile, Bokhman group, FIGO stage, LVSI, depth of myometrial invasion, T2DM status and treatment received.

### HALP scores and endometrial cancer overall, cancer-specific and recurrence-free survival

HALP scores ranged from 4.7 to 115.6 with a median score of 37.6 and IQR of 26.0–50.2. There was a significant but weak correlation between HALP scores and BMI (Spearman correlation coefficient 0.106, p = 0.026). There was no evidence of a correlation between HALP and age (Spearman correlation coefficient 0.03, p = 0.476). A prognostic cut-off of 24 was selected based on previous publications [31]. A total of 95 women (21.6%) had HALP scores <24 and were classed as having ‘low’ HALP scores whilst the remaining 344(78.4%) with scores of at least 24 were classed as having ‘high’ HALP scores. There was evidence of a significant association between HALP scores and FIGO stage (p = 0.005), histology (p = 0.037), disease grade (p = 0.020), LVSI (p = 0.027), myometrial invasion (0.001), recurrence rates (0.044) and mortality status (p = 0.026) (Table 2). There was no evidence for an association with age, BMI, T2DM, social deprivation quintile or treatment received.

Next, we evaluated the performance of HALP as a continuous variable in predicting overall, cancer-specific and recurrence-free survival. The unadjusted and adjusted hazard ratios for overall mortality were 0.99(95% CI 0.98–1.00, p = 0.113) and 0.99 (95%CI 0.98–1.01, p = 0.368), respectively. For cancer-specific mortality, the hazard ratios were 0.99(95%CI 0.97–1.00, p = 0.152) and 1.00(95%CI 0.99–1.01, p = 0.925), respectively, while for disease recurrence, the unadjusted and adjusted hazard ratios were 0.99(95%CI 0.98–1.01, p = 0.229) and 1.00(95%CI 0.99–1.02, p = 0.647).

Next, we assessed the performance of HALP prognostic categories based on a cut-off of 24 [31]. The 5-year survival rates were 67% (55–77%) and 79% (72–84%) for women with low and high HALP scores respectively. Women with high HALP scores had reduced overall and cancer-specific mortality rates compared to those with low HALP scores in univariable analyses (Fig 1B, Table 4). Crude hazard ratios were 0.56 (95% CI 0.36–0.88, p = 0.012-) and 0.50 (95% CI 0.30–0.84, p = 0.009) for overall and cancer-specific mortality risks, respectively (Table 4). In addition, women with high HALP scores had lower recurrence rates (unadjusted HR 0.53, 95% CI 0.310.88, p = 0.015) than those with low HALP scores (Table 4). Following adjustment for clinico-pathological factors, however, there was no evidence for an effect of HALP on overall, cancer-specific or recurrence-free survival (Table 4).

**Table 4:** Cox regression analysis of haemoglobin, albumin, lymphocyte and platelet (HALP) score categories and endometrial cancer survival outcomes with crude and adjusted hazard ratios and 95% confidence intervals.

Categories	Unadjusted HR(95% CI)	p-value	Adjusted HR(95%CI)	p-value
**Overall Mortality**				
HALP <24	1.00		1.00	
HALP ≥24	0.56 (0.36–0.88)	**0.012**	0.71 (0.44–1.17)	0.182
**Cancer-Specific Mortality**				
HALP <24	1.00		1.00	
HALP ≥24	0.50 (0.30–0.84)	**0.009**	0.72 (0.40–1.29)	0.274
**Disease Recurrence**				
HALP <24	1.00		1.00	
HALP ≥24	0.53 (0.31–0.88)	**0.015**	0.69 (0.39–1.21)	0.196

Adjusted model includes age, BMI, socioeconomic quintile, Bokhman group, FIGO stage, LVSI, depth of myometrial invasion, T2DM status and treatment received.

## Discussion

### Main findings

This was a prospective cohort study of 439 endometrial cancer patients followed up for a median duration of 42 months. In this study, we show evidence for the potential utility of PNI as a prognostic biomarker in endometrial cancer. Women with elevated pre-treatment PNI at a decision threshold of 45 had a 45% decrease in overall and endometrial cancer-specific mortality risk compared to those with low PNI. There was no evidence for an association between PNI and recurrence free survival. We found an association between HALP scores and aggressive tumour parameters, specifically FIGO stage, histology, disease grade, LVSI and deep myometrial invasion, but when these and clinical prognostic factors were controlled for, there was no evidence that HALP scores are associated with overall, cancer-specific or recurrence-free survival.

### Strengths and limitations

To our knowledge, this is the first study to explore the impact of PNI and HALP on endometrial cancer survival outcomes. We analysed data from a large prospective cohort of endometrial cancer patients recruited to various population-based studies that posed no restriction according to clinico-pathological parameters, thus alleviating concerns about selection bias. The availability of data on relevant tumour clinico-pathological factors allowed for robust adjustment for confounding and effect modification. Well known endometrial cancer prognostic factors including FIGO stage, disease grade, histological subtype, LVSI and depth of myometrial invasion, were consistent in demonstrating prognostic associations with endometrial cancer. Whilst PNI and HALP showed an association with endometrial cancer survival outcomes in univariable and/or multivariable analyses, they are non-specific scores that may be affected by unmeasured confounders that we could not account for. The lack of data on the TCGA molecular classification of endometrial cancer may have led to residual confounding and an over- or under-estimation of survival outcomes. The generally favourable prognosis of endometrial cancer and the consequent low-event rate affects the precision of our estimates. We obtained haemoglobin, albumin, lymphocyte and platelet counts pre-treatment, but not at later time points, and as such, we are unable to comment on the dynamic change of PNI and HALP at the various stages of management. Finally, as this was a single centre prospective study of mainly White British women, we are unable to generalise our study findings to women from other centres, nationalities or ethnic groups.

### Interpretation

Host immunity and nutritional status are strong predictors of outcomes in cancer patients [7, 11, 32]. Circulating immune inflammatory cells including lymphocytes, neutrophils and platelets have been implicated in the proliferation, invasion, and metastasis of malignant cells [11]. Lymphocytes, including T cells and natural killer cells, are part of the body’s cell-mediated immunity and are an important defence against cancers [32, 33]. Cytotoxic lymphocytes and natural killer cells are the key players in cancer immune-surveillance and activate programmed intracellular events, ultimately leading to apoptotic death of tumour cells [32]. Serum albumin, the most abundant protein in human serum, is a negative acute phase reactant that is often used as a surrogate marker for nutritional status [34]. The meta-analysis by Cabrerizo and colleagues found serum albumin to be a good marker of nutritional status in clinically stable older patients [35]. However, its utility is limited by its lack of specificity and long half-life [36] A low serum albumin level has been shown to correlate with survival outcomes in a wide range of malignancies [37, 38], including those of the endometrium [39, 40]. Weight loss, changes in BMI and decease in muscle mass are other surrogate markers of nutritional status. However, their utility in endometrial cancer is limited as most women with endometrial cancer are obese and weight loss is recommended [35, 41]. The consensus is therefore that laboratory parameters, including albumin derived indices, should be used alongside a thorough physical examination when assessing nutritional status [42]. Several immune-nutritional and inflammatory parameters have been suggested as potential endometrial cancer prognostic biomarkers, including the Naples prognostic score, controlling nutritional status (CONUT) score, neutrophil-to-lymphocyte ratio, monocyte-to-lymphocyte ratio, platelet-to-lymphocyte ratio, Glasgow prognostic score and C-reactive protein [43–45]. However, the evidence to enable their clinical translation is limited.

PNI is a composite measure of the immune-nutritional status and a promising cancer prognostic biomarker. The computation of PNI is based on a linear predictive model that incorporates pre-treatment lymphocyte and albumin counts [17]. PNI was originally developed by Onodera as a risk marker for post-operative complications following gastrointestinal surgery [46]. Subsequent studies have been consistent in demonstrating a correlation between low PNI values and poor prognosis in various tumours including those of the ovary [9], lung [10], breast [47] and colon [19]. The meta-analysis by Dai and colleagues investigated the impact of PNI on treatment outcomes in 2050 women with ovarian cancer who were surgically managed and concluded that patients with a low PNI had shorter overall and progression-free survival compared to those with a high PNI [9]. A low PNI was also found to correlate with FIGO stage, presence of ascites, larger residual tumour volume, poor response to chemotherapy, and higher levels of CA-125 [9]. However, this review was limited by marked heterogeneity across the included studies and a dominance of studies from Japan and China, thus raising concerns about the generalisability of the study findings. The meta-analysis by Wang and colleagues, involving 2372 patients with gynaecological malignancies concluded that PNI closely correlates with both overall and progression-free survival [21]. However, no endometrial cancer studies were included in this review. Our study provides evidence for the potential prognostic utility of PNI in endometrial cancer. PNI is a simple and cost-effective measure of immune-nutritional status. If validated in a larger independent cohort, PNI has the potential to refine endometrial cancer pre-treatment risk assessment. A low PNI was recorded for almost 20% of women, for whom immune and nutrition based interventions and careful post-treatment follow-up may be justified. “Can a blood test predict survivorship and/or recurrent disease?” ranked 5th most important endometrial cancer research priority by a James Lind Alliance Partnership representing views of patients, carers, and clinician groups [48]. A prognostic blood test, therefore, has strong appeal for women with endometrial cancer and clinicians alike.

The HALP score is a comprehensive index of the nutritional and immune status of patients and has been reported to correlate with survival outcomes in cancers of the gastrointestinal [49] and genitourinary tracts [50]. HALP integrates haematological parameters, specifically haemoglobin, albumin, lymphocyte and platelet counts. A low haemoglobin count reflects anaemia, a common finding in cancer patients. Cancer-associated anaemia results from chronic blood loss, nutritional deficiency (iron and vitamins) and advanced stage malignancy [51]. Inflammation and malnutrition both lead to a depletion of albumin while a low lymphocyte count may represent impaired host immune-surveillance [12, 32]. Platelets modulate tumour angiogenesis via platelet-derived substances including microRNA and various surface receptors [52, 53]. Our finding of a correlation between HALP and adverse clinico-pathological parameters is consistent with those reported for other tumour sites [23, 54]. Peng and colleagues reported an association between low HALP scores and older age, female sex, high tumour stage, high American Society of Anesthesiology (ASA) grade and anaemia, based on a retrospective analysis of 516 patients treated for bladder cancer with radical cystectomy [23]. A similar study of 1360 patients with renal cell carcinoma treated with nephrectomy found that a low HALP score strongly correlated with being female, older age, a high Fuhrman grade, high TNM stage, presence of sarcomatoid transformation, tumour necrosis and lymphovascular space invasion [54]. In our study, there was an association between HALP and FIGO stage, histology, disease grade and deep myometrial invasion, but when these and clinical prognostic factors were controlled for, there was no evidence that HALP scores correlate with overall, cancer-specific or recurrence-free survival at the suggested prognostic cut-off value of 28. Data-derived prognostic thresholds vary widely across studies, and markers like HALP are yet to have a clinically validated prognostic cut-off. It is therefore possible that a different conclusion might be reached based on alternative thresholds. Well-designed endometrial cancer prognostic studies with adequate sample sizes are now needed to confirm the true value of HALP and to identify optimal decision thresholds.

## Conclusion

In this study, we found pre-treatment PNI to be an independent prognostic biomarker in endometrial cancer. Women with elevated PNI at a prognostic cut-off value of 45 had a 45% decrease in mortality risk compared to women with low PNI. If validated in a larger independent cohort, PNI could serve as a simple, cost-effective prognostic parameter with potential to improve endometrial cancer pre-operative risk assessment. HALP scores, on the other hand, were associated with adverse clinico-pathologic factors, but not overall, cancer-specific or recurrence-free survival in multivariable analyses.
